# Ferritin is associated with the severity of lung involvement but not with worse prognosis in patients with COVID-19: data from two Italian COVID-19 units

**DOI:** 10.1038/s41598-021-83831-8

**Published:** 2021-03-01

**Authors:** Francesco Carubbi, Lia Salvati, Alessia Alunno, Fabio Maggi, Erika Borghi, Rinalda Mariani, Francesca Mai, Maurizio Paoloni, Claudio Ferri, Giovambattista Desideri, Sabrina Cicogna, Davide Grassi

**Affiliations:** 1 Internal Medicine and Nephrology Unit, Department of Life, Health and Environmental Sciences, University of L’Aquila, San Salvatore Hospital, L’Aquila, Italy; 2 Department of Medicine, ASL 1 Avezzano-Sulmona-L’Aquila, San Salvatore Hospital, L’Aquila, Italy; 3 Infectious Diseases Unit, Department of Medicine, Ospedale SS Filippo e Nicola, ASL 1 Avezzano-Sulmona-L’Aquila, L’Aquila, Italy; 4 Department of Medicine, University of Perugia, Perugia, Italy; 5 Department of Radiology, Ospedale SS Filippo e Nicola, ASL 1 Avezzano-Sulmona-L’Aquila, L’Aquila, Italy

**Keywords:** Biomarkers, Diseases, Infectious diseases, Viral infection

## Abstract

The coronavirus 2019 disease (COVID-19) is characterised by a heterogeneous clinical presentation, a complex pathophysiology and a wide range of imaging findings, depending on disease severity and time course. We conducted a retrospective evaluation of hospitalized patients with proven SARS-CoV-2 infection, clinical signs of COVID-19 and computed tomography (CT) scan-proven pulmonary involvement, in order to identify relationships between clinical, serological, imaging data and disease outcomes in patients with COVID-19. Clinical and serological records of patients admitted to two COVID-19 Units of the Abruzzo region in Italy with proven SARS-CoV-2 pulmonary involvement investigated with CT scan, assessed at the time of admission to the hospital, were retrospectively evaluated. Sixty-one patients (22 females and 39 males) of median age 65 years were enrolled. Fifty-six patients were discharged while death occurred in 5 patients. None of the lung abnormalities detected by CT was different between discharged and deceased patients. No differences were observed in the features and extent of pulmonary involvement according to age and gender. Logistic regression analysis with age and gender as covariates demonstrated that ferritin levels over the 25th percentile were associated with the involvement of all 5 pulmonary lobes (OR = 14.5, 95% CI 2.3–90.9, p = 0.004), the presence of septal thickening (OR = 8.2, 95% CI 1.6–40.9, p = 0.011) and the presence of mediastinal lymph node enlargement (OR = 12.0, 95% CI 1.1–127.5, p = 0.039) independently of age and gender. We demonstrated that ferritin levels over the 25th percentile are associated with a more severe pulmonary involvement, independently of age and gender and not associated with disease outcomes. The identification of reliable biomarkers in patients with COVID-19 may help guiding clinical decision, tailoring therapeutic approaches and ultimately improving the care and prognosis of patients with this disease.

## Introduction

In late 2019, an outbreak of viral pneumonia caused by a novel coronavirus, the severe acute respiratory syndrome coronavirus 2 (SARS-CoV-2) was identified in Wuhan, China^1^; subsequently, the coronavirus 2019 disease (COVID-19) spread worldwide, and on March 11, 2020, the WHO declared COVID-19 a global pandemic^2^.

COVID-19 clinical presentation ranges from asymptomatic infection to interstitial pneumonia, respiratory failure, acute respiratory distress syndrome (ARDS) and sepsis^3^. The pathophysiology is complex, involving immune and hematologic systems, epithelial cells and vascular system^4^. Several pro-inflammatory cytokines and chemokines, interleukin (IL)-1β, IL-2, IL-6, IL-7, IL-10, tumour necrosis factor-α (TNFα), granulocyte colony-stimulating factor (G-CSF), interferon (IFN) γ-induced protein 10 (IP-10/CXCL10), monocyte chemoattractant protein 1 (MCP-1), macrophage inflammatory protein 1α (MIP-1α/CCL3), have been detected in the bloodstream and target tissue of COVID-19 patients^1,3,5^. Severe lymphopenia is a peculiar feature of patients with severe disease since the majority of patients with mild disease present without this manifestation^6–8^. The inflammatory cytokine storm is likely one of the main factors behind the observed lymphopenia. The serum level of pro-inflammatory cytokines, such as TNF-α and IL-6, have been closely correlated with lymphopenia, while recovered patients show close to normal levels of such cytokines. Importantly, severe lymphopenia predisposes to ICU stay^6^ and mortality, where during hospitalization, non‐survivors demonstrated a more significant deterioration in lymphopenia compared with those who survived^7,8^.

In more detail, T lymphocytes including CD4^+ ^and CD8^+^ subtypes and NK cells are reduced in patients with severe disease^9^. Conversely, monocytes and macrophages are increased, explaining elevated levels of some pro-inflammatory cytokines. All this fits with the evidence that the predominant pattern of lung lesions in patients with COVID-19 patients is a diffuse alveolar damage, with the presence of platelet–fibrin thrombi in small arterial vessels, and the great majority of the inflammatory cells infiltrating the lungs are monocytes, macrophages and CD4^+^ lymphocytes^10,11^.

Further events lead to activation of the coagulation cascade through endothelial and tissue factor pathways, paralleling the systemic inflammation^12, 13^. Moreover, some Authors suggested that COVID-19 might be considered as a peculiar cardiovascular (CV) disease based on predisposing patients to arterial and venous thrombosis and particularly as consequence of hypercoagulability^14,15^ and endothelial dysfunction^16,17^. Indeed, it has been reported that CV complications are quickly emerging as a key threat in COVID-19 beyond respiratory involvement. The mechanisms underlying the disproportionate effect of SARS-CoV-2 infection on patients with CV comorbidities are not yet fully elucidated. Nevertheless, Iaccarino et al. recently showed that in patients with COVID-19 mortality is predicted by age and comorbidities, particularly diabetes mellitus, chronic obstructive pulmonary disease, and chronic kidney disease but not hypertension^18^.

The massive release of pro-inflammatory mediators and the aberrant activation of the immune and coagulation systems, resembles the so-called cytokine release syndrome, a group of conditions sharing the same pathogenic mechanism, although with a different aetiology^19^. This cytokine storm accounts for the two main causes of mortality in COVID-19, ARDS and secondary haemophagocytic lymphohistiocytosis, the latter occurring in a small subset of patients^20^. Furthermore, since increased levels of ferritin along with a cytokine storm have been described in patients with severe COVID-19^21^, it has been speculated that COVID-19 may be included in the spectrum of the hyperferritinemic syndromes^20^.

COVID-19 pneumonia displays a wide range of imaging findings, depending on disease severity and time course, and can overlap with a variety of infectious and non-infectious pulmonary diseases. The choice of imaging in COVID-19 is left to the judgement of clinical teams at the point-of-care accounting for the differing attributes of X-ray and computed tomography (CT), local resources, and expertise^22^. However, CT is more sensitive for early parenchymal lung disease, disease progression, and alternative diagnoses including acute heart failure from COVID-19 myocardial injury and pulmonary thromboembolism^23^. Typically, COVID-19 pneumonia is characterised by bilateral peripheral patchy ground-glass opacities (GGO) with or without consolidation; superimposed interlobular septal thickening can also be present, resulting in a crazy-paving pattern^24,25^. Pleural effusion or septal thickening have been rarely described^26^, while recently an Italian study described lymphadenopathy in up to 58% of cases^27^. GGO alone, followed by GGO with consolidation, is the most frequent finding in mild cases, while in severe cases with ARDS widespread dense consolidative opacification are present^23^.

The aim of this study was to describe clinical, serological and CT imaging features of a cohort of patients with COVID-19 pneumonia and identify possible relationships between the variables and disease outcomes (admission to intensive care unit (ICU) and/or death).

## Methods

### Patient cohort and data collection

Clinical and serological records of patients admitted to two COVID-19 Units of the Abruzzo region in Italy (S. Salvatore Hospital, L’Aquila and SS Filippo e Nicola Hospital, Avezzano) with proven SARS-CoV-2 pulmonary involvement investigated with CT scan, assessed at the time of admission to the hospital, were retrospectively evaluated. All patients presented the clinical and laboratory signs of COVID-19 infection, as assessed by positive SARS-CoV-2 RT-PCR testing.

Collected data included: age, gender, days of symptoms before the CT scan, red blood cell count, hemoglobin (Hb), platelet (PLT) count, white blood cell (WBC) count, absolute neutrophil count (ANC), absolute lymphocyte count (ALC), absolute monocyte count (AMC), lymphocyte subpopulation (CD3^+^, CD4^+^, CD8^+^, CD16^+^, CD19^+^), erythrocyte sedimentation rate (ESR), C-reactive protein (CRP), ferritin, D-dimer, fibrinogen, lactate dehydrogenase (LDH), procalcitonin, arterial oxygen partial pressure:fractional inspired oxygen ratio (PaO_2_/FiO_2_). Neutrophils:lymphocytes (N:L) ratio and ferritin:ESR ratio were calculated based on the available data. Anemia was defined according to the WHO criteria as Hb values below 12 g/dl in females and 13 g/dl in males^28^. All the other cytopenias were defined according to the reference levels of the local laboratory (leukopenia: WBC < 4.00 × 10^3^/μL; neutropenia: ANC < 2.00 × 10^3^/μL; lymphocytopenia: ALC < 0.80 × 10^3^/μL).

All patients underwent chest CT and the images were examined by an independent radiologist (FB) with more than 10-year experience on chest CT imaging. The information collected were: lobes involved and presence/absence of: GGOs, parenchymal consolidation, septal thickening, pleural effusion, mediastinal lymph node enlargement^29^. Two semi-quantitative scoring systems were used to quantify the extension of the disease: i. each of the 5 lobes (right upper lobe, right middle lobe, right lower lobe, left upper lobe and left lower lobe) was scored on a scale of 0–3 (0: no lesion, 1: < 1/3 of the lobe volume involved, 2: > 1/3 and < 2/3 of the lobe volume involved, 3: > 2/3 of the lobe volume involved) (score A)^30^; ii. each of the five lung lobes was assessed for degree of involvement and classified as none (0%), minimal (1–25%), mild (26–50%), moderate (51–75%), or severe (76–100%). No involvement corresponded to a lobe score of 0, minimal involvement to a lobe score of 1, mild involvement to a lobe score of 2, moderate involvement to a lobe score of 3, and severe involvement to a lobe score of 4 (Score B)^31^. An overall lung “total severity score” was reached by summing the five lobe scores (Score A, range of possible scores, 0–15; Score B range of possible scores, 0–20).

All methods were carried out in accordance with Good Clinical Practice guidelines and all patients provided written informed consent in accordance with the declaration of Helsinki. Ethical review and approval (ASL1 Avezzano-Sulmona-L’Aquila) was obtained in accordance with local legislation and institutional requirements.

### Statistical analysis

Data were analyzed with STATA/SE 16.1. The Mann Whitney U test was used to compare continuous variables while the χ^2^ and Fisher’s exact tests were used as needed for categorical variables. Bivariate correlation (Spearman’s ρ) and binary logistic regression analysis were also performed. All tests were two tailed and values of p < 0.05 were considered statistically significant.

## Results

### Characteristics of the study cohort

Sixty-one patients (22 females and 39 males) with a median age of 65 years (range 32–93 years) were enrolled (Table 1). Sixty percent of patients displayed at least 2 comorbidities, and one quarter of the overall cohort had four or more comorbidities. Upon admission to the COVID-19 ward patients received supportive therapy, including supplemental oxygen. None of the patients received immunomodulatory treatment (including but not limited to corticosteroids prior the CT scan). Acute phase reactants were abnormal in the majority of patients while with regard to blood cell counts, 26/60 patients (43%) did not display any cytopenia. Anemia was detectable in 22/60 patients (37%), leukopenia in 9 patients (15%) and thrombocytopenia in 11/60 patients (18%). In 27/60 patients (45%) only one cytopenia was present, in 6/60 patients (10%) cytopenia involved 2 cell lines, in only 1 patient involved all the 3 cell lines. A reduction of ANC was observed in 6/60 patients (10%) while ALC was below the lower limit in 15/60 patients (25%). Of interest, 7/60 patients (12%) displayed lymphocytosis. The mean N:L ratio was 5.07. The ferritin value was available in 51 out of 61 patients and increased in 38 of them (74%). The average time between the onset of respiratory symptoms and the chest CT was 5.69 days. Overall, pulmonary involvement was moderate to severe, with 59% of subjects having 5 lobes involved and 58 patients (95%) displaying bilateral disease. About half of the patients displayed only GGO and the other half both GGO and consolidation. Pleural effusion was observed only in a small number of patients (N = 11; 18%) while 46 patients (75%) displayed septal thickening. Mediastinal lymph node enlargement was detected in 20 (33%) of patients. Mean PaO_2_/FiO_2_ value was 300.1, with only 5/58 (9%) patients displaying normal values (> 400), 23/58 (40%) patients displaying slight impairment (300–400); 24/58 (41%) patients displaying moderate impairment (200–300) and 6/58 (10%) patients displaying severe impairment. The average values of the two CT severity scores were 5.85 and 6.69 respectively. Table 2 shows the correlations between pulmonary involvement and different serological variables. As far as the disease outcomes are concerned, 56 patients (92%) were discharged while 5 patients (8%) deceased. Of the 56 discharged patients, five (9%) required admission to ICU before discharge.

**Table 1:** Clinical, serological and computed tomography scan features of the study cohort (N = 61).

	Median (range)
Age (years)	65 (32–93)
Days of symptoms before CT	5 (1–16)
WBC (N × 10^3^/μL)	5.54 (2.33–13.14)
ANC (N × 10^3^/μL)	3.73 (1.17–11.15)
ALC (N × 10^3^/μL)	1.07 (0.48–2.49)
AMC (N × 10^3^/μL)	0.38 (0.13–1.33)
N:L ratio	3.52 (0.88–21.0)
CD3^+^ cells (N × 10^3^/μL)	756 (288–1941)
CD4^+^ cells (N × 10^3^/μL)	446 (194–1188)
CD8^+^ cells (N × 10^3^/μL)	322 (53–1025)
CD19^+^ cells (N × 10^3^/μL)	112 (35–224)
CD16^+^ cells (N × 10^3^/μL)	172 (32–777)
CD4^+^/CD8^+^ ratio	1.80 (0.60–9.30)
Hb (g/L)	13 (8.80–17.70)
PLTs	203.50 (92–444)
ESR (mm 1 h)	67 (2–116)
CRP (mg/dl)	4.15 (0.03–18.80)
Ferritin	549 (23.40–5031.60)
Ferritin:ESR ratio	7.79 (0.32–213.20)
D-dimer	1 (0.27–18.30)
Fibrinogen	555 (228–1021)
LDH	300 (96–998)
Procalcitonin	0.09 (0.02–1.97)
PaO_2_/FiO_2_	229.50 (95–523)
Severity ScoreA	6 (1–13)
Severity Score B	6 (1–18)
**Gender**	N (%)
F	22 (36)
M	39 (64)
**Number of comorbidities**	
0	11 (18)
1	14 (23)
2	14 (23)
3	7 (11)
4	6 (10)
5 or more	9 (15)
**No. of lobes affected**	
1	2 (3)
2	7 (11)
3	7 (11)
4	9 (15)
5	36 (59)
Bilateral lung disease	58 (95)
**Type of parenchymal lesions**	
Neither ground glass opacities nor consolidation	0 (0)
Ground glass opacities only	32 (52)
Consolidation only	0 (0)
Ground glass opacities and consolidation	29 (48)
Septal thickening	46 (75)
Pleural effusion	11 (18)
Mediastinal lymph node enlargement	20 (33)
**Outcome**	
Discharged	56 (92)
With ICU admission	5 (9)
Without ICU admission	51 (91)
Deceased	5 (8)

*CT* computed tomography, *WBC* white blood cells, *ANC* absolute neutrophil count, *ALC* absolute lymphocyte count, *AMC* absolute monocyte count, *N:L* neutrophils:lymphocytes, *Hb* haemoglobin, *PLT* platelets, *ESR* erythrocyte sedimentation rate, *CRP* C reactive protein, *LDH* lactate dehydrogenase, *ICU* intensive care unit.

**Table 2:** Correlations between pulmonary involvement and serological variables.

	Variable	Spearman’s ρ	p value
PaO_2_/FiO_2_	ANC	− 0.325	0.013
	N:L ratio	− 0.375	0.004
	CRP	− 0.303	0.022
	D-dimer	− 0.268	0.048
	LDH	− 0.504	< 0.0001
	Procalcitonin	− 0.395	0.003
	Severity score A	− 0.506	< 0.0001
	Severity score B	− 0.518	< 0.0001
CT Severity score A	ANC	0.305	0.017
	ALC	− 0.486	< 0.0001
	N:L ratio	0.496	< 0.0001
	CD3^+^ cells	− 0.559	< 0.0001
	CD4^+^ cells	− 0.531	0.001
	CD8^+^ cells	− 0.430	0.008
	CD16^+^ cells	− 0.328	0.048
	ESR	0.298	0.032
	CRP mg/dl	0.532	< 0.0001
	Ferritin	0.529	< 0.0001
	D-dimer	0.278	0.035
	Fibrinogen	0.517	< 0.0001
	LDH	0.518	< 0.0001
	Procalcitonin	0.366	0.005
CT Severity score B	ANC	0.298	0.020
	ALC	− 0.460	< 0.0001
	N:L ratio	0.470	< 0.0001
	CD3^+^ cells	− 0.526	0.001
	CD4^+^ cells	− 0.491	0.002
	CD8^+^ cells	− 0.404	0.013
	CD16^+^ cells	− 0.331	0.045
	ESR	0.324	0.019
	CRP	0.473	< 0.0001
	Ferritin	0.548	< 0.0001
	Ferritin:ESR	0.305	0.047
	Fibrinogen	0.504	< 0.0001
	LDH	0.564	< 0.0001
	Procalcitonin	0.349	0.008

*CT* computed tomography, *ANC* absolute neutrophil count, *ALC* absolute lymphocyte count, *AMC* absolute monocyte count, *N:L* neutrophils:lymphocytes, *Hb* haemoglobin, *PLT* platelets, *ESR* erythrocyte sedimentation rate, *CRP* C reactive protein, *LDH* lactate dehydrogenase.

### Subgroup analysis by age and gender

When patients were divided according to the age (below or over 65 years, Table 3) a few variables resulted significantly different. In particular, lower CD8 lymphocyte counts and lower Hb levels along with higher CRP and D-dimer levels were observed in patients over 65 years (all p values < 0.05). As expected by lower Hb values, anemia was more prevalent in patients over the age of 65 (p = 0.032). Of interest, while the prevalence of thrombocytopenia, leukopenia and lymphopenia was similar in the 2 groups, all patients with neutropenia were below the age of 65 (p = 0.009) and all but one patient with lymphocytosis were over the age of 65 (p = 0.05). In addition, the N:L ratio was significantly higher in patients over 65 years of age (p = 0.03). To note, neither the features and extent of pulmonary involvement, nor the functional impairment seemed to be influenced by age of onset. Likewise, no difference in the features and extent of pulmonary involvement, nor in the functional impairment emerged comparing males to females. However, males displayed significantly higher levels of ferritin and, as a consequence, of the ferritin:ESR ratio.

**Table 3:** Differences across groups dividing patients according to age and gender.

	< 65 years old	≥ 65 years old	p value^1^	Females	Males	p value^1^
	Median (range)	Median (range)		Median (range)	Median (range)	
N (%)	30 (49)	31 (51)		22 (36)	39 (64)	
Age (years)	56.5 (32–64)	78 (65–93)	< 0.0001	75 (32–90)	64 (39–93)	ns
Days of symptoms before CT	6 (1–11)	5 (1–16)	ns	5 (1–16)	5 (1–11)	ns
WBC (N × 10^3^/μL)	4.98 (2.33–12.97)	6.1 (3.76–13.14)	ns	4.78 (2.41–12.45)	5.94 (2.33–13.14)	ns
ANC (N × 10^3^/μL)	3.29 (1.17–11.04)	4.1 (2.30–11.15)	ns	3.20 (1.37–11.15)	3.84 (1.17–11.04)	ns
ALC (N × 10^3^/μL)	0.11 (0.48–02.16)	0.96 (0.48–2.49)	ns	1.12 (0.48–1.71)	1.06 (0.48–2.49)	ns
AMC (N × 10^3^/μL)	0.34 (0.13–0.82)	0.46 (0.17–1.33)	ns	0.39 (0.17–0.82)	0.37 (0.13–1.33)	ns
N:L ratio	3.16 (0.89–16.70)	4.36 (1.02–21.16)	0.027	3.34 (1.15–18.27	3.85 (0.89–21.16)	ns
CD3 ^+^ cells (N × 10^3^/μL)	978 (468–1941)	643 (288–1837)	ns	653.50 (421–1825)	776 (288–1941)	ns
CD4^ +^ cells (N × 10^3^/μL)	546 (251–1188)	410.50 (194–912)	ns	477 (251–1188)	420 (194–894)	ns
CD8^ +^ cells (N × 10^3^/μL)	388 (53–1017)	236.50 (83–1025)	0.048	246.50 (53–629)	354 (89–1025)	ns
CD19^ +^ cells (N × 10^3^/μL)	143 (38–224)	82.50 (35–214)	ns	108.50 (47–214)	112 (35–224)	ns
CD16^ +^ cells (N × 10^3^/μL)	152 (64–374)	176 (32–777)	ns	180 (32–335)	152 (36–777)	ns
CD4^+^/CD8^+^ ratio	1.60 (0.60–9.30)	1.90 (0.7–5.1)	ns	2.05 (0.60–9.30)	1.60 (0.70–4.10)	0.01
Hb (g/L)	13.75 (10.3–16.0)	12.45 (8.8–17.7)	0.015	12.55 (9.70–14.40)	13.80 (8.80–17.70)	0.003
PLTs	190 (92–444)	239.50 (110–373)	ns	233 (110–395)	190 (92–444)	ns
ESR (mm/h)	67 (2–114)	68 (30–116)	ns	76 (11–112)	61.00 (2.00–116.00)	ns
CRP (mg/dl)	2.06 (0.03–17.30)	4.64 (0.04–18.8)	ns	3.43 (0.03–18.80)	4.72 (0.04–17.30)	ns
Ferritin	539.75 (27.0–5031.6)	590.5 (23.4–3876.2)	ns	281.95 (23.40–705.90)	760.30 (34–5031)	< 0.0001
Ferritin:ESR ratio	10.28 (0.3–213.2)	7.02 (0.6–88.1)	ns	3.92 (0.32–27.83)	13.46 (1.37–213.20)	0.002
D-dimer	0.72 (0.33–2.48)	1.35 (0.27–18.30)	0.005	1.23 (0.27–4.00)	0.94 (0.34–18.30)	ns
Fibrinogen	529 (271–1021)	556 (228–775)	ns	555 (228–775)	556.50 (371–1021)	ns
LDH	299 (96–514)	301 (135–998)	ns	269 (135–732)	312 (96–998)	ns
Procalcitonin	0.07 (0.02–1.31)	0.12 (0.02–1.97)	ns	0.07 (0.02–1.97)	0.10 (0.02–1.31)	ns
PaO_2_/FiO_2_	309 (95–523)	293 (122–500)	ns	314 (214–523)	289 (95–424)	ns
Severity score A	5 (1–10)	6 (1–13)	ns	5.50 (1–10)	6.00 (1.00–13.00)	ns
Severity score B	6 (1–12)	6 (1–18)	ns	5.50 (1–12)	7.00 (1.00–18.00)	ns

	< 65 years old	≥ 65 years old	p value^2^	Females	Males	p value^2^
	N (%)	N (%)		N (%)	N (%)	
**Gender**			ns			< 0.0001
F	9 (30)	13 (42)		22 (100)	0 (0)	
M	21 (70)	18 (58)		0 (0)	39 (100)	
**No. of lobes affected**			ns			ns
1	1 (3)	1 (3)		2 (9)	0 (0)	
2	3 (10)	4 (13)		2 (9)	5 (13)	
3	5 (17)	2 (6)		3 (14)	4 (10)	
4	4 (13)	5 (16)		4 (18)	5 (13)	
5	17 (57)	19 (61)		11 (50)	25 (64)	
Bilateral lung disease	28 (93)	30 (97)	ns	20 (91)	38 (97)	ns
**Type of parenchymal lesions**			ns			ns
Neither ground glass opacities nor consolidation	0 (0)	0 (0)		0 (0)	0 (0)	
Ground glass opacities only	17 (57)	15 (48)		9 (9)	23 (59)	
Consolidation only	0 (0)	0 (0)		0 (0)	0 (0)	
Ground glass opacities and consolidation	13 (43)	16 (52)		13 (59)	16 (41)	
Septal thickening	21 (70)	25 (81)	ns	15 (68)	31 (79)	ns
Pleural effusion	4 (13)	7 (23)	ns	6 (27)	5 (13)	ns
Mediastinal lymph node enlargement	9 (30)	11 (35)	ns	9 (41)	11 (28)	ns

*CT* computed tomography, *WBC* white blood cells, *ANC* absolute neutrophil count, *ALC* absolute lymphocyte count, *AMC* absolute monocyte count, *N:L* neutrophils:lymphocytes, *Hb* haemoglobin, *PLT* platelets, *ESR* erythrocyte sedimentation rate, *CRP* C reactive protein, *LDH* lactate dehydrogenase.

^1^p value calculated with Mann Whitney U test.

^2^p value calculated with the χ2 test or the Fisher's exact test as needed.

### Subgroup analysis by ferritin levels

No difference in the extent or severity of pulmonary manifestations was observed. We divided the 51 patients with available ferritin values according to percentiles (Table 4) rather than using the reference level of the local laboratory to ensure generalizability of the results. Patients with ferritin values over the 25^th^ percentile were more frequently males and also displayed higher levels of fibrinogen, LDH and procalcitonin compared to those with ferritin levels below the 25^th^ percentile. To note, the extent of pulmonary involvement (number of lobes affected, bilateral involvement, and severity scores) as well as the prevalence of septal thickening and mediastinal lymph node enlargement were significantly higher in patients with higher levels of ferritin while the functional impairment (PAO_2_/FiO_2_) was more pronounced in this group.

**Table 4:** Differences across groups dividing patients according to ferritin levels.

	Ferritin below 25th percentile	Ferritin over 25th percentile	p value^1^
	Median (range)	Median (range)	
N (%)	13/51 (25)	38/51 (75)	
Age	64 (32–89)	64.50 (39–93)	ns
Days of symptoms before CT	5 (1–10)	5 (1–16)	ns
WBC (N × 10^3^/μL)	4 (2.41–12.45)	5.7400 (2.33–13.14)	ns
ANC (N × 10^3^/μL)	2 (1.37–10.87)	3.8600 (1.17–11.04)	0.01
ALC (N × 10^3^/μL)	1 (0.72–2.25)	1.0600 (.48–2.49)	0.02
AMC (N × 10^3^/μL)	(.17-.82)	0.38 (0.13–1.33)	ns
N:L ratio	1.79 (1.02–9.29)	3.8 (1.09–21.16)	0.002
CD3^ +^ cells (N × 10^3^/μL)	1087.50 (421–1825)	722.00 (288–1941)	ns
CD4^ +^ cells (N × 10^3^/μL)	639.50 (251–1188)	416.00 (194–894)	ns
CD8^ +^ cells (N × 10^3^/μL)	394.50 (120–715)	322.00 (53–1025)	ns
CD19^ +^ cells (N × 10^3^/μL)	137 (44–208)	86.00 (35–224)	ns
CD16^ +^ cells (N × 10^3^/μL)	205.50 (86–364)	152.00 (36–777)	ns
CD4^+^/CD8^+^ ratio	1.9 (0.6–2.5)	1.60 (0.7–9.3)	ns
Hb (g/L)	11.80 (10.3–14.2)	13.70 (8.80–17.70)	0.04
PLTs	231 (139–373)	193 (92–444)	ns
ESR (mm/h)	61 (11–108)	71 (2–116)	ns
CRP (mg/dl)	0.93 (0.03–11.60)	4.8100 (0.37–17.30)	0.01
D-dimer	0.59 (0.35–4.00)	1.04 (0.27–18.30)	ns
Fibrinogen	426 (271–627)	557.00 (228–1021)	0.01
LDH	209 (163–438)	325.00 (135–998)	0.01
Procalcitonin	0.02 (0.02–0.79)	0.12 (0.02–1.31)	0.0003
PaO_2_/FiO_2_	340 (214–523)	290.00 (95–500)	0.02
Severity scoreA	3 (1–6)	6.00 (2–13)	0.0004
Severity score B	3 (1–7)	7.00 (2–18)	0.0004

	Ferritin below 25th percentile	Ferritin over 25th percentile	p value^2^
	N (%)	N (%)	
**Gender**			0.003
F	9 (69)	9 (24)	
M	4 (31)	29 (76)	
**No. of lobes affected**			< 0.0001
1	2 (15)	0 (0)	
2	5 (38)	2 (5)	
3	1 (8)	5 (13)	
4	3 (23)	4 (11)	
5	2 (15)	27 (71)	
Bilateral lung disease	10 (77)	38 (100)	0.002
**Type of parenchymal lesions**			ns
Neither ground glass opacities nor consolidation	0 (0)	0 (0)	
Ground glass opacities only	8 (62)	19 (50)	
Consolidation only	0 (0)	0 (0)	
Ground glass opacities and consolidation	5 (39)	19 (50)	
Septal thickening	5 (38)	32 (84)	0.001
Pleural effusion	5 (38)	4 (11)	0.02
Mediastinal lymph node enlargement	1 (8)	14 (37)	0.05

*CT* computed tomography, *WBC* white blood cells, *ANC* absolute neutrophil count, *ALC* absolute lymphocyte count, *AMC* absolute monocyte count, *N:L* neutrophils:lymphocytes, *Hb* haemoglobin, *PLT* platelets, *ESR* erythrocyte sedimentation rate, *CRP* C reactive protein, *LDH* lactate dehydrogenase, *M* male, *F* female.

^1^p value calculated with Mann Whitney U test.

^2^p value calculated with the χ2 test or the Fisher's exact test as needed.

We subsequently performed logistic regression analysis and since older age and male gender have been identified as predictors of more severe disease in previous studies and ferritin was significantly higher in males in our cohort, we included both age and gender as covariates. Ferritin levels over the 25^th^ percentile were associated with the involvement of all 5 pulmonary lobes (Odds ratio (OR) = 14.5, 95% CI 2.3–90.9, p = 0.004), the presence of septal thickening (OR = 8.2, 95% CI 1.6–40.9, p = 0.011) and the presence of mediastinal lymph node enlargement (OR = 12.0, 95% CI 1.1–127.5, p = 0.039) independently of age and gender. The N:L ratio was also associated with the presence of septal thickening (OR = 2.0, 95% CI 1.2–3.5, p = 0.008) but at multivariate analysis only ferritin remained significantly associated with this pulmonary manifestation (OR = 5.1, 95% CI 1.1–24.3, p = 0.043).

### Subgroup analysis by disease outcomes

When comparing discharged patients according to their need to be admitted to ICU before discharge, we observed significantly higher severity scores and significantly lower PAO_2_/FiO_2_ values in patients needing ICU, as well as higher D-dimer and LDH values (Table 5). However, none of the other variables was significantly different between the groups. When comparing discharged and deceased patients it emerged that deceased patients were older, with higher WBC and ANC counts and higher N:L ratios along with higher LDH and procalcitonin levels and lower PaO_2_/FiO_2_ values. None of the lung abnormalities detected by CT was different between discharged and deceased patients.

**Table 5:** Characteristics of patients according to the outcome.

	Discharged			Deceased (N = 5), Median (range)	p value discharged vs deceased^1^	p value discharged with vs without ICU admission^1^
	All (N = 56)	With ICU admission (N = 5)	Without ICU admission (N = 51)			
	Median (range)	Median (range)	Median (range)			
Age	63.50 (32–93)	69 (58–78)	62 (32–93)	78 (65–91)	0.039	ns
Days of symptoms before CT	5 (1–16)	4 (1–9)	6 (1–16)	5 (1–7)	ns	ns
WBC (N × 10^3^/μL)	5.18 (2.33–13.14)	6.06 (4.65–13.14)	5.07 (2.33–12.97)	11.78 (4.84–12.45)	0.023	ns
ANC (N × 10^3^/μL)	3.37 (1.17–11.04)	4.30 (2.93–10.16)	3.32 (1.17–11.04)	10.16 (3.70–11.15)	0.005	ns
ALC (N × 10^3^/μL)	1.07 (0.48–2.49)	1.27 (0.48–2.14)	1.07 (0.48–2.49)	0.83 (0.55–1.17)	ns	ns
AMC (N × 10^3^/μL)	0.380 (0.13–1.33)	0.81 (0.20–1.33)	0.38 (0.13–0.82)	0.30 (0.24–1.06)	ns	ns
N:L ratio	3.28 (0.89–21.16)	4.32 (2.45–21.16)	3.21 (0.89–16.71)	9.29 (4.30–18.47)	0.004	ns
Hb (g/L)	13.30 (9.70–16.70)	13.20 (11.40–16.0)	13.3 (9.70–16.70)	12.10 (8.8–17.7)	ns	ns
PLTs	203 (92–444)	187.50 (167–314)	203 (92–444)	261 (125–373)	ns	ns
ESR (mm/h)	67 (2–116)	48 (30–67)	67 (2–116)	86 (44–94)	ns	ns
CRP (mg/dl)	3.55 (0.03–17.30)	5.10 (3.55–13.98)	2.99 (0.03–17.30)	9.03 (3.55–18.80)	ns	ns
Ferritin	539.75 (23.4–5031.60)	796 (530.50–1750.10)	473.50 (23.40–5031.60)	629.30 (213.20–876.20)	ns	ns
Ferritin:ESR ratio	7.79 (0.3–213.2)	12.30 (7.9–53.9)	7.28 (0.30–213.20)	47. 39 (6.70–88.10)	ns	ns
D-dimer	0.91 (0.27–18.30)	1.94 (0.99–11.88)	0.85 (0.27–18.30)	1.48 (0.65–4.00)	ns	0.037
Fibrinogen	552 (228–1021)	552 (458–767)	542 (228–1021)	595.50 (498–627)	ns	ns
LDH	297 (96–534)	362 (301–534)	277.50 (96–514)	732 (379–998)	0.009	0.044
procalcitonin	0.086 (0.02–1.31)	0.33 (0.04–0.57)	0.08 (0.02–1.31)	0.66 (0.10–1.97)	0.015	ns
PaO_2_/FiO_2_	313 (95–523)	196 (122–278)	320.50 (95–523)	223 (133–280)	0.008	0.002
Severity score A	6 (1–11)	10 (2–11)	6 (1–10)	8 (2–13)	ns	0.022
Severity score B	6 (1–13)	12 (2–13)	6 (1–12)	10 (2–18)	ns	0.033

	Discharged			Deceased (N = 5), N (%)	p value discharged vs deceased^2^	p value discharged with vs without ICU admission^2^
	All (N = 56)	With ICU admission (N = 5)	Without ICU admission (N = 51)			
	N (%)	N (%)	N (%)			
**Gender**					ns	ns
F	20 (36)	0 (0)	20 (39)	2 (40)		
M	36 (64)	5 (100)	31 (61)	3 (60)		
Age over 65 years	26 (46)	4 (80)	22 (43)	5 (100)	ns	ns
**No. of lobes affected**					ns	ns
1	2 (4)	0 (0)	2 (4)	0 (0)		
2	6 (11)	1 (20)	5 (10)	1 (20)		
3	7 (13)	0 (0)	7 (14)	0 (0)		
4	7 (13)	1 (20)	6 (12)	2 (40)		
5	34 (61)	3 (60)	31 (61)	2 (40)		
Bilateral lung disease	53 (95)	5 (100)	48 (94)	5 (100)	ns	ns
**Type of parenchymal lesions**						
Neither ground glass opacities nor consolidation	0 (0)	0 (0)	0 (0)	0 (0)		
Ground glass opacities only	31 (55)	2 (40)	29 (57)	1 (20)		
Consolidation only	0 (0)	0 (0)	0 (0)	0 (0)		
Ground glass opacities and consolidation	25 (45)	3 (60)	22 (43)	4 (80)		
Septal thickening	41 (73)	4 (80)	37 (73)	5 (100)	ns	ns
Pleural effusion	9 (16)	1 (20)	8 (16)	2 (40)	ns	ns
Mediastinal lymph node enlargement	18 (32)	1 (20)	17 (33)	2 (40)	ns	ns

*CT* computer tomography, *ICU* intensive care unit, *SD* standard deviation, *WBC* white blood cells, *ANC* absolute neutrophil count, *ALC* absolute lymphocyte count, *AMC* absolute monocyte count, *N:L* neutrophils:lymphocytes, *Hb* haemoglobin, *PLT* platelets, *ESR* erythrocyte sedimentation rate, *CRP* C reactive protein, *LDH* lactate dehydrogenase, *M* male, *F* female.

^1^p value calculated wiith Mann Whitney U test.

^2^p value calculated with the χ^2^ test or the Fisher's exact test as needed.

## Discussion

Although in the majority of patients COVID-19 is characterized by mild to moderate symptoms, it is burdened by consistent morbidity and mortality due to respiratory failure, ARDS and sepsis^3^.

In our cohort the prognosis was rather good despite the severity of pulmonary involvement, with only a small number of subjects needing to be transferred to ICU and/or eventually deceased. In addition, we demonstrated and quantified for the first time a strong association between ferritin levels and the severity of pulmonary involvement independently of age and gender.

Since December 2019 an increasing number of articles describing the clinical, serological, radiological and histological features of patients with COVID-19 across the world have been published^32^. Of interest, in addition to chest X-ray and CT scan, lung ultrasonography has been pointed as a cost-effective non-invasive approach to assess pulmonary involvement in the scenario of a pandemic^33,34^.

Italy has been early and dramatically affected by COVID-19 although with a large diversity across the Country^35^. The Abruzzo region is within the 10 less affected regions per number of cases but with a relatively high case fatality ratio of 13%. However, despite the severity of the pulmonary involvement, the overall prognosis in our cohort was good with most of the patients requiring ICU being subsequently discharged and only 8% patients deceased. Furthermore, our data regarding the prevalence of different pulmonary lesions in COVID-19 partially fit with the previous literature^36,37^.

It has been speculated that pulmonary oedema and hyaline membrane formation may be the underlying pathological driver of GGO sign^38,39^. GGO may develop into reticular interlobular septa thickening and crazy paving pattern, indicating that the infection leads to diffuse alveolar oedema and interstitial inflammation^40^. Of interest, septal thickening was the most frequent lesion in our cohort observed in 75% of patient. Multifocal, patchy, or segmental consolidation, distributed in subpleural areas or along bronchovascular bundles, is usually detected in the 2–64% of COVID-19 pneumonia^26,37^ and may relate to cellular fibromyxoid exudates in alveoli^38, 39^. Several data suggest that the presence of consolidation and its increase overtime could be an indicator of disease progression^40–42^, however this was not the case in our cohort. We confirmed that pleural effusion is a rare imaging finding and none of our patients displayed coexisting lymphadenopathy. Based on the experience of other viral pneumonia such as influenza and other coronavirus diseases, the presence of pleural effusion may suggest a poor prognosis in COVID-19 patients^37,38,42–47^ and if coexisting with lymphadenopathy bacterial superinfection or another diagnosis should be ruled out^36,38^. Among demographic and serological features, several parameters, such as older age and male gender, have been associated with more severe COVID-19 prognosis^18^. Furthermore, higher ferritin levels have been described in patients with more severe disease and deceased^21,48–50^.

These findings fit with the concept of hyperinflammation in COVID-19, and since hyperferritinemia has been associated with inflammatory states in SARS-CoV-2 infection, it is plausible that ferritin may be a useful parameter to predict disease severity and the extent of the cytokine storm.

Ferritin has been well characterised as an acute phase reactant, as well as a mediator of immune dysregulation in severe COVID-19^51^. Therefore, ferritin may be not only a marker of the inflammatory milieu but also an active player in the "cytokine storm" scenario that characterizes severe COVID-19. Complex feedback mechanisms between ferritin and cytokines in the control of pro-inflammatory and anti-inflammatory mediators might exist as cytokines can induce ferritin expression, but ferritin can induce the expression of pro- and anti-inflammatory cytokines as well. In this regard, it has been speculated that COVID-19 with pulmonary involvement may be included within the spectrum of hyperferritinemic syndrome^20,52^.

In our cohort, age was not related to ferritin levels but male patients displayed significantly higher levels. Of interest, however, when stratifying patients according to the levels of ferritin, the association between higher ferritin levels and a more sever clinical picture independently of age and gender became evident. Hyperferritinemic syndromes share clinical and serological features such as fever, cytopenias, multiorgan involvement and coagulopathy^53^, most of which can also be observed in COVID-19. In fact, despite a lung-centric immunopathology, currently available data suggest that the clinical spectrum of COVID-19 is not limited to lung involvement, but rather represents a multisystem illness^4^. Patients with higher ferritin levels displayed a higher N:L ratio, which has been associated with more severe pulmonary involvement in COVID-19^54,55^. In addition, higher D-dimer levels, hinting coagulation abnormalities, were also detected in COVID-19 patients with higher ferritin levels. The most intriguing finding is that ferritin was not associated with the disease outcome (discharge, admission to ICU or death) and neither was D-dimer, the latter in contrast with a retrospective Chinese study^3^. It is therefore tempting to speculate that ferritin may have a pathogenic role in the development of lung damage in COVID-19 but the exact mechanisms need to be fully elucidated. Inflammation is an essential part of an effective immune response and it has also clearly been indicated as a specific promoter of endothelial dysfunction and enhancer of the atherosclerotic process with increasing the CV risk^56^. Since SARS-CoV-2 can induce excessive and prolonged cytokine/chemokine responses that may eventually lead to death, the identification of reliable biomarkers enabling early stratification of patients developing cytokine storm and at which extent is warranted. This would also allow tailoring of immunomodulatory treatment strategies. This bulk of evidence may also support the “endothelial” hypothesis with the thrombotic or thromboembolic implications COVID-19-dependent associated to the cytokine storm^57,58^.

We acknowledge that our study displays some limitations, such as its retrospective nature, the small number of patients and the lack of pro-inflammatory cytokine levels and follow-up data.

## Conclusions

We demonstrated for the first time that ferritin levels over the 25^th^ percentile are associated with severe pulmonary involvement as detected by CT scan but not with the disease outcome. We also confirmed that CT findings of GGO with or without consolidation are the earliest and common visible manifestation of COVID-19 pneumonia, highlighting its central role in the diagnosis and severity evaluation of the disease. An accurate assessment of radiologic and pathologic features in COVID-19 patients is warranted to gain further knowledge on disease pathogenesis, clinical course and prognosis. This, along with the implementation of reliable biomarkers of lung disease severity in COVID-19 patients will allow early diagnosis, tailored treatment and ultimately improve patient care.
